# Bone metastases from bladder perivascular epithelioid cell tumor – an unusual localization of a rare tumor: a case report

**DOI:** 10.1186/1752-1947-8-227

**Published:** 2014-06-25

**Authors:** Giovanni Palleschi, Antonio Luigi Pastore, Salvatore Evangelista, Luigi Silvestri, Luigi Rossi, Claudio Di Cristofano, Natale Porta, Vincenzo Petrozza, Silverio Tomao, Antonio Carbone

**Affiliations:** 1 Department of Biotechnologies and Medico-Surgical Sciences, Urology Unit, ICOT, Sapienza University of Rome, Latina, Italy; 2 Uroresearch® Association, Latina, Italy; 3 Department of Biotechnologies and Medico-Surgical Sciences, Oncology Unit, ICOT, Sapienza University of Rome, Latina, Italy; 4 Department of Biotechnologies and Medico-Surgical Sciences, Histopathology Unit, ICOT, Sapienza University of Rome, Latina, Italy; 5 Department of Medical and Surgical Sciences and Biotechnologies, Unit of Urology, Faculty of Pharmacy and Medicine, Sapienza University of Rome, Corso della Repubblica 79, 04100 Latina, LT, Italy

**Keywords:** Bladder, Chemotherapy, Metastasis, Perivascular epithelioid cell tumors

## Abstract

**Introduction:**

Perivascular epithelioid cell tumors are mesenchymal tumors composed of histologically and immunohistochemically distinctive perivascular epithelioid cells. This type of tumor is rare but bladder localization is even rarer.

**Case presentation:**

A case of bone metastatic bladder perivascular epithelioid cell tumor in a 65-year-old Caucasian man treated with surgery and chemotherapy is described and compared with other reports in the literature.

**Conclusions:**

The rarity of perivascular epithelioid cell tumors hinders the development of a standard therapeutic approach, and thus requires case report descriptions. There is a need for cooperative studies to enlarge the case series and establish the best treatment strategy for this rare disease.

## Introduction

The World Health Organization describes perivascular epithelioid cell tumors (PEComas) as “mesenchymal tumors composed of histologically and immunohistochemically distinctive perivascular epithelioid cells” [1]. The PEComa family of tumors includes angiomyolipoma, clear cell “sugar” tumor, lymphangioleiomyomatosis, and numerous unusual visceral, intra-abdominal, soft tissue, and bone tumors. These have a variety of names, including clear cell myomelanocytic tumor of the falciform ligament/*ligamentum teres*, abdominopelvic sarcoma of perivascular epithelioid cells, and primary extrapulmonary sugar tumor, among others [2].

PEComas are rare and only 13 cases of bladder localization have been reported in the literature [3]. We describe a case of skeletal metastatic PEComa arising from the urinary bladder.

## Case presentation

**Figure 1:**
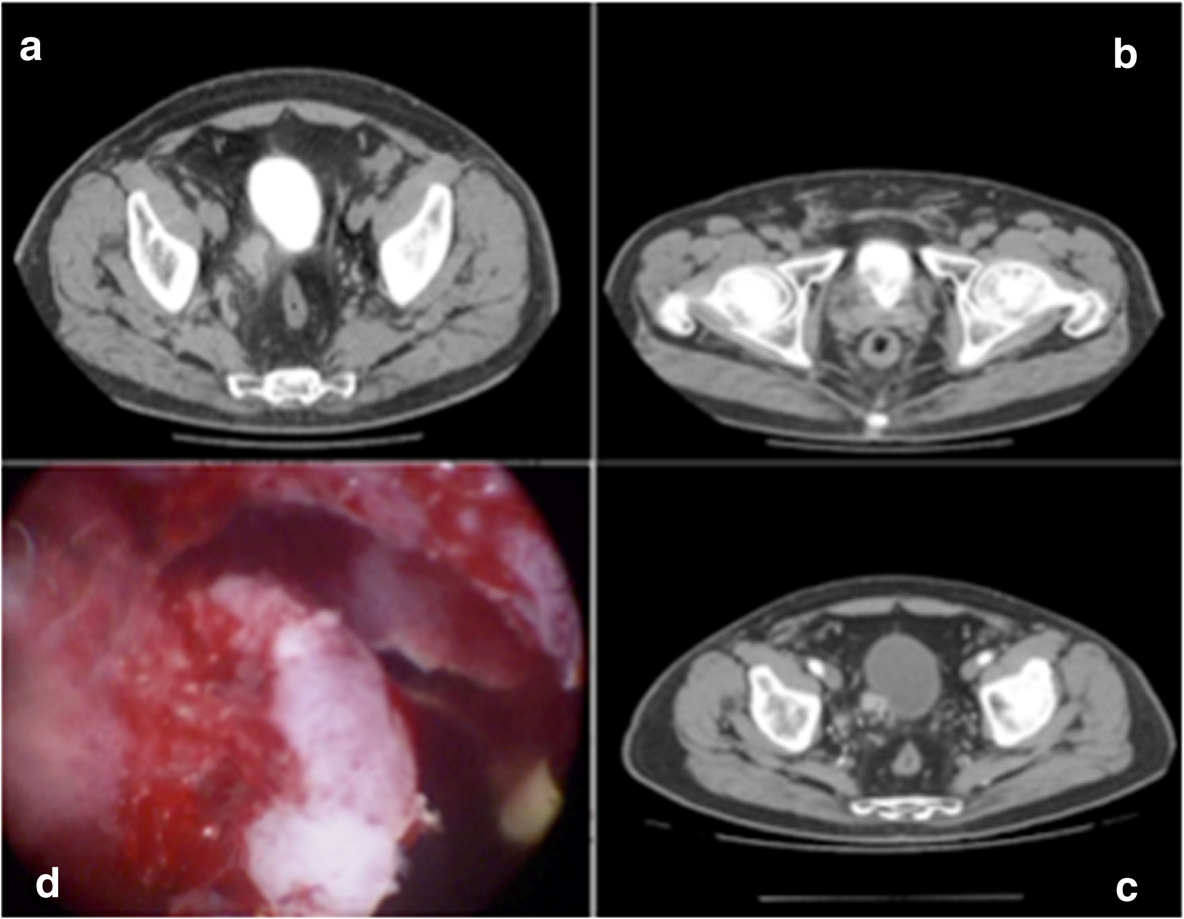
**Computed tomography and transurethral resection of the bladder lesion.** CT showed mild heterogeneous enhancement of the lesion, which infiltrated the bladder wall**,** no other lesions or local or systemic adenopathy were observed **(a–c)**. Transurethral resection of bladder **(d)**.

**Figure 2:**
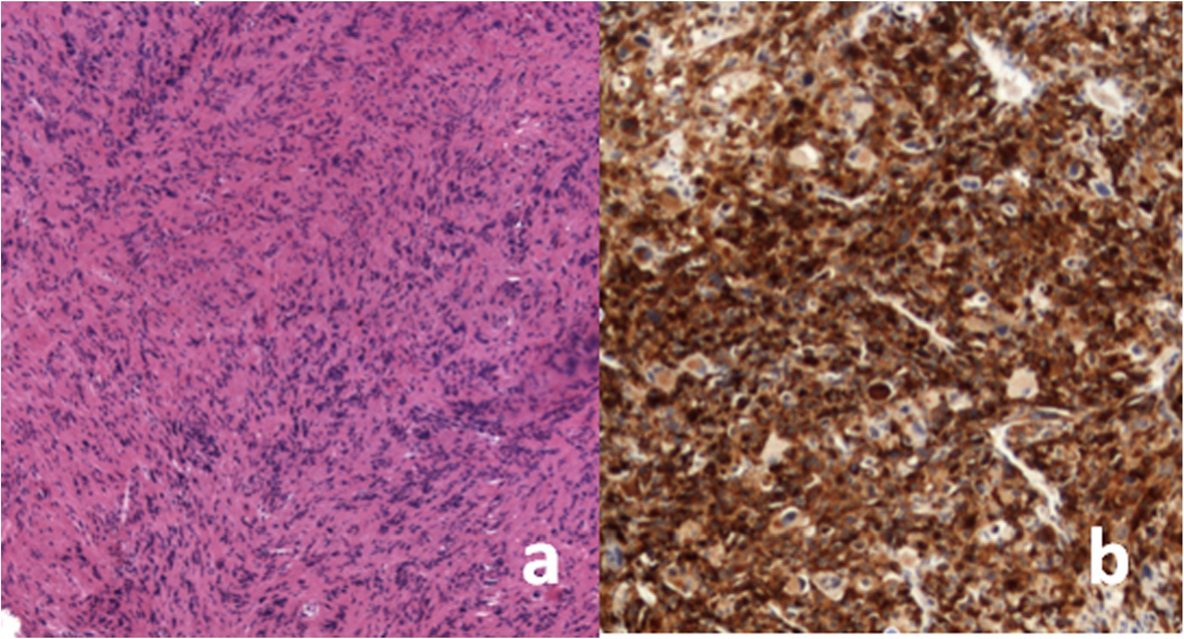
Microscopic examination (magnification 10×) showing a mixture of epithelioid and spindle-shaped cells with prominent blood vessels (a) and strong positivity for human melanoma black-45 at immunohistochemistry (b).

A 65-year-old Caucasian man (body mass index 25kg/m^2^) had a history of ischemic heart disease and lower urinary tract symptoms secondary to benign prostatic hyperplasia, which was under pharmacological treatment with alpha-blockers. He consulted us due to recurrent hematuria and back pain that had been unsuccessfully treated with antibiotics and anti-inflammatory drugs in the previous 2 months. His physical examination was normal. A digital rectal examination revealed a moderate increase in prostatic gland size without any finding suspicious for neoplastic disease.The results of his blood tests were normal and his total prostate-specific antigen value was 2.2ng/mL. An abdominal ultrasound showed normal kidneys and a lesion of his right bladder wall associated with a 2cm calculus. On cystoscopy, his bladder lesion was a solid, sessile, mass of 2.5cm on the right lateral wall, 3cm from his right ureteral orifice; a 2cm bladder stone was confirmed. Abdominopelvic contrast computed tomography (CT) showed mild heterogeneous enhancement of the lesion, which infiltrated his bladder wall. No other lesions or local or systemic adenopathy were observed (Figure 1a–c). He then underwent endoscopic removal of the mass and a simultaneous transurethral cystolithotripsy (Figure 1d).No intra- and postoperative complications were observed; he was discharged on the second postoperative day after catheter removal. Histopathology of the lesion showed spindled and epithelioid clear neoplastic cells with prominent blood vessels (Figure 2a); immunohistochemistry was positive for human melanoma black-45 and negative for S-100, vimentin, smooth muscle actin, cytokeratin, and desmin (Figure 2b). The tissue exhibited an infiltrative growth pattern with high mitotic activity (more than 1/50 high-power field) and necrosis. These findings were suggestive of a malignant PEComa.

Based on the histopathological examination and negative CT findings with symptoms (persistent back pain), positron emission tomography (PET) was performed, which showed an increased uptake of the radiopharmaceutical drug at specific skeletal segments: somas of L1, L5, and the left iliac wing. After surgery, he had a performance status of 1 and reported a moderate pain in his lumbar region with a visual analog scale (VAS; from 1 to 10) of 5. Chemotherapy was therefore planned. Previous ischemic heart disease and the echocardiographic finding of reduced systolic function with first-degree diastolic dysfunction prevented the use of anthracyclines, which are the standard first-line treatment for soft tissue sarcomas [4-10]. Gemcitabine was therefore administered, starting with a 30-minute infusion of 1000mg/m^2^ on days 1, 8, and 15 of a 4-week cycle. He was clinically re-evaluated before each dose. His performance status did not change during the treatment and pain in his lumbar region disappeared after the third administration (VAS: 1). The treatment was well tolerated and analgesics were not necessary. Side effects were nausea, asthenia, moderate thrombocytopenia (90,000 platelet count/mcL) and neutropenia (4100 white blood cells/mcL).

Six months after starting chemotherapy, CT assessment showed a stable disease according to the response evaluation criteria in solid tumors; no bladder recurrence was observed on PET or cystoscopic control; skeletal metastases were reduced and no other secondary lesions were observed. He is presently clinically stable.

## Discussion

The definition of PEComa was suggested by Zamboni *et al.* in 1996 to identify a group of mesenchymal neoplasms originating from perivascular epithelioid cells [11]. However, only a few cases of urinary bladder PEComa have been reported in the literature; fewer with bone metastasis [12]. We therefore consider our case to be of interest. The diagnosis of PEComa is based only on histopathological examination because there are no macroscopic features or imaging details that allow identification before surgical removal, which is mandatory. Furthermore, given the rarity of these tumors, there is no specific consensus regarding the ideal therapy after surgery in case of bladder and other localizations. It is still controversial whether these tumors can be considered benign or if they all carry some intrinsic risk of aggressive behavior. In this regard, PEComas with a major risk of progression are described by the Folpe criteria as those >5cm, with infiltrative behavior, high nuclear grade, high cellularity, high mitotic rate, presence of necrosis, and vascular invasion [13].

Based on this classification, adding medical treatment to surgery in patients with these histopathologic findings is advisable. Unfortunately, the authors who reported their experience of PEComas used different therapies and reported different follow-up procedures, thus preventing comparison of outcomes and assessment of a standard protocol for treatment [4]. Specifically considering experience with bladder PEComas, all authors report surgical removal as the first step in treatment (endoscopic bladder resection, or partial or radical cystectomy), but postoperative medical management is still poorly reported.

In the present case, gemcitabine was selected as chemotherapy due to the patient’s cardiovascular disease, which did not allow the use of drugs with cardiotoxic risk. At present, he is clinically stable with no cystoscopic, PET, or CT evidence of disease progression, thus implying good efficacy of our therapeutic approach. Conversely, Parfitt *et al.* treated a bladder PEComa with primary excision and adjuvant interferon-alpha immunotherapy with evidence of remission until 48 months of follow-up [14]. No other adjuvant therapies specific for bladder localization are described in literature to the best of our knowledge.

In terms of medical treatment, even though not specifically for bladder localization, encouraging outcomes have been reported with administration of mammalian target of rapamycin (mTor) inhibitors. In particular, a case of retroperitoneal PEComa was reported in a man with complete disease regression after treatment with everolimus [15]. PEComas are related to the genetic alterations of tuberous sclerosis complex (TSC), an autosomal dominant genetic disease associated with losses of *TSC1* (9q34) or *TSC2* (16p13.3) genes, which seem to have a role in the regulation of the Rheb/mTOR/p70S6K pathway.

However, as reported by Martignoni *et al.*, it should be noted that these approaches derive from anecdotal cases as no therapeutic trial has so far been implemented due to the rarity of the disease [16]. An international cooperative study would enlarge the case series and help to address this problem.

## Conclusions

The majority of bladder tumors are of urothelial origin and, generally, small lesions are considered to have limited metastatic risk. However, the case reported here shows that the histopathological characteristics of a bladder tumor may sometimes be different and carry a high metastatic risk.

PEComas are very rare, but they may occur in the urinary bladder; the case reported here adds to the small body of literature that demonstrates that they can metastasize in different sites of the human body. Surgical removal remains the most important therapeutic approach while controversy is still ongoing about the best postoperative therapeutic management. Based on the large body of experience present in the literature on the treatment of soft tissue tumors, the use of anthracyclines could also be considered a good therapeutic option for PEComas, but single-agent chemotherapy with gemcitabine should be considered an option in patients selected for comorbidities until standard treatment protocols can be generated from larger comparative trials.

## Consent

Written informed consent was obtained from the patient for publication of this case report and any accompanying images. A copy of the written consent is available for review by the Editor-in-Chief of this journal.

## Abbreviations

CT: Computed tomography; PEComas: Perivascular epithelioid cell tumors; PET: Positron emission tomography; TSC: Tuberous sclerosis complex; VAS: Visual analog scale.

## Competing interests

The authors declare that they have no competing interests.

## Authors’ contributions

GP and ALP conceived the study and design. GP, ALP, LS, LR, CDC, NP, SE, VP, ST, and AC undertook acquisition of data. GP, ALP, LS and SE analyzed and interpreted the data and drafted the manuscript. SE, LR, and NP performed critical revision of the manuscript. AC, ALP, ST supervised the study. All authors read and approved the final manuscript.
